# LifeWatch Greece data-services: Discovering Biodiversity Data using Semantic Web Technologies

**DOI:** 10.3897/BDJ.4.e8443

**Published:** 2016-11-01

**Authors:** Nikos Minadakis, Yannis Marketakis, Martin Doerr, Chryssoula Bekiari, Panagiotis Papadakos, Alexandros Gougousis, Nicolas Bailly, Christos Arvanitidis

**Affiliations:** ‡ FORTH - Institute of Computer Science, Heraklion, Crete, Greece; § HCMR, Heraklion, Crete, Greece

**Keywords:** Data Integration, Biodiversity Metadata, Data Discovery, Semantic Web

## Abstract

**Background:**

Biodiversity data is characterized by its cross-disciplinary character, the extremely broad range of data types and structures, and the variety of semantic concepts that it encompasses. Furthermore there is a plethora of different data sources providing resources for the same piece of information in a heterogeneous way. Even if we restrict our attention to Greek biodiversity domain, it is easy to see that biodiversity data remains unconnected and widely distributed among different sources.

**New information:**

To cope with these issues, in the context of the LifeWatch Greece project, i) we supported cataloguing and publishing of all the relevant metadata information of the Greek biodiversity domain, ii) we integrated data from heterogeneous sources by supporting the definitions of appropriate models, iii) we provided means for efficiently discovering biodiversity data of interest and iv) we enabled the answering of complex queries that could not be answered from the individual sources. This work has been exploited, evaluated and scientificaly confirmed by the biodiversity community through the services provided by the LifeWatch Greece portal.

## Introduction

One of the main characteristics of biodiversity data is its cross-disciplinary character and the extremely broad range of data types, structures, and semantic concepts that it encompasses. Even if we focus on the data of a specific area (i.e. Greece), they remain widely distributed and unconnected. For example occurrence records in csv format may be stored in the University of Thessaloniki, dna sequencing fasta files can be stored in the University of Patras and MicroCT scanning images may be stored in the HCMR's databases.

For this purpose our idea was to: (a) extract information about the events that are explicitly or implicitly contained in the datasets, (b) model the different concepts and entities of the biodiversity domain and (c) take advantage of the semantic graph’s capabilities to navigate efficiently through the different contents and discover data of interest. This approach provides the means of making high level, abstract, queries without having full knowledge about the domain, the schema or even the presence of the data, and easily discovering data resources that contain (or are) the results of such queries.

**Figure 1.**
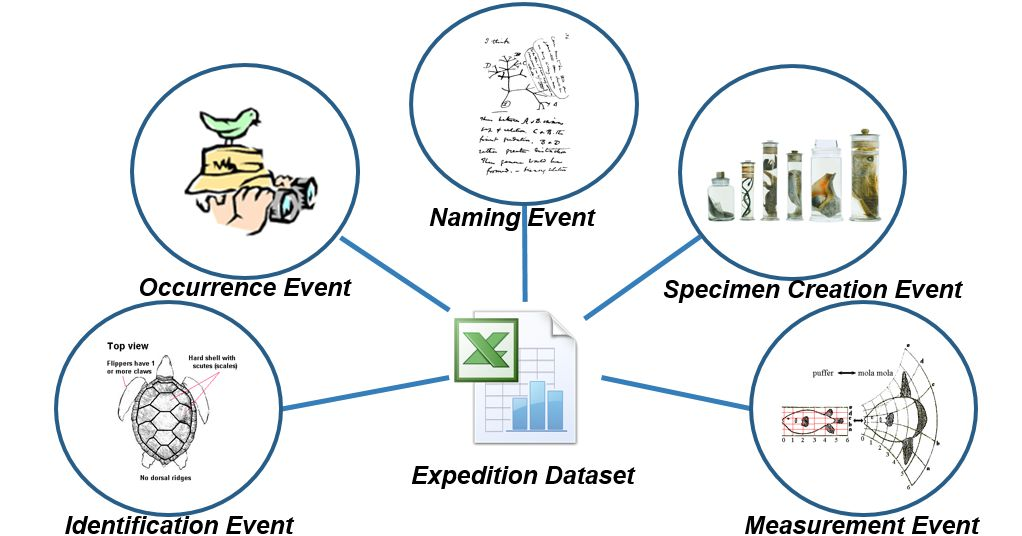
Events that are being described in an Expendition Dataset

As regards the extraction of information about events, it is evident that an expedition dataset contains information about a number of sub-events such as occurrence or identification events (Fig. 1). So, apart from the metadata of the dataset that can be stored in a registry, information about the sub-events can be extracted, modeled and used to assist the discovery of data resources.

In the context of LifeWatchGreece Research Infrastructure we designed and implemented a set of data services that aim to: i) support cataloguing and publishing all the relevant metadata information of the Greek biodiversity domain, ii) integrate data from heterogeneous sources by supporting the definitions of appropriate models, iii) efficiently discover biodiversity data of interest and enable the answering of complex queries that could not be answered from the individual sources. The aforementioned services allow the providers to express their metadata in a schema agnostic way; the provider is able to submit metadata according to their local format (e.g. Darwin Core) and these are automatically transformed with respect to the underlying centralized schemata of the infrastructure for gaining the advantages that semantic models offer. Particular focus was given on the architecture and the contents of the infrastructure for being able to serve clients, even if some of its parts are temporarily inaccessible.

## Project description

### Design description

The activities have been split in three different phases, namely: the requirements/specification phase, the design phase and the implementation phase. Below we will describe each one of them in details along with the web application that has been developed and a real usage scenario example that demonstrates the data services capabilities.

**Requirements/Specification Phase**

The requirements/specification phase contained all the activities as regards the data analysis and collection of requirements. Specifically, the first step of the requirements/specification phase was the definition of the functional requirements of the infrastructure.

After the collection and the analysis of the functional requirements it was clear that there was a need for creating a number of e-Services that will assist the Data Providers to submit their metadata and data to the LifewatchGreece Research Infrastructure by providing functionalities of: a) **transformation** of the metadata from the provider’s schema to the centralized semantic schema of the infrastructure, b) **ingestion** of the metadata and data to LifewatchGreece Research Infrastructure, c) **publishing** of the datasets by providing information on how to access the datasets and how to communicate with the datasets’ creator/curator, d) **querying** in order to gain information on how to access the datasets and how to communicate with the curator/creator, e) **quality improvement** of the datasets that the Data Providers will publish to the infrastructure. The next step was to design the data workflow based on the Synergy Reference Model for Data Provisioning and Aggregation.

To evaluate the representation needs of the biodiversity domain it was essential to collect and analyze a significant number of datasets coming from the community. More than 50 datasets coming from different sections of the broader biodiversity domain were collected. Following this activity, the collected datasets were analyzed, categorized and the mandatory metadata were identified and extracted.

The step that followed the collection and analysis of the datasets was the collection of the competency queries. These queries are derived from the domain experts, express the final expectation of the user from the system and are used for defining the main functional requirements as well as the final evaluation of the services. They are essential for the design and the evaluation of the ontology and the definition of the main scientific questions that should be answered. More than 70 competency queries were collected from the domain experts. An indicative example of a competency query is:

*Find all taxonomic names and their associated authorities that have ever been placed in genus X (even if now they are in another genus), including information on the locality, habitat and depth / elevation of the type specimen for each of those species*.

Moreover, the analysis of the Greek biodiversity community’s datasets and metadata led to the ***identification of fourteen (14) main metadata categories*** than can cover the full set of metadata that should be kept in the metadata catalogue. These categories are:

- *Occurrences*
- *Identifications*
- *Taxonomy*
- *Naming*
- *MicroCT Scannings*
- *Environmental*
- *Specimens*
- *Specimen Collections*
- *Data Collections*
- *Genetics*
- *Morphometrics*
- *Morphological Characteristics*
- *Statistical*
- *Occurrence Statistics*

A Metadata Catalogue was created, documenting for each metadata category a set of mandatory metadata. In order to avoid duplications and assist the data integration process we decided to model these metadata with respect to a set of semantic models. Specifically, we designed a new model for the purposes of the LifeWatch Greece project that was inspired from CIDOC CRM [Doerr 2003​], and its extensions CRM dig [Theodoridou et al. 2010, CRM sci, CMR geo and MarineTLO [Tzitzikas et al. 2013][Tzitzikas et al. 2016]. The catalogue contains more than 200 metadata fields descriptions, 300 examples, and more than 100 mappings to the semantic models. A few mapping examples between DwC and our models is shown in Table 1.

**Figure 2.**
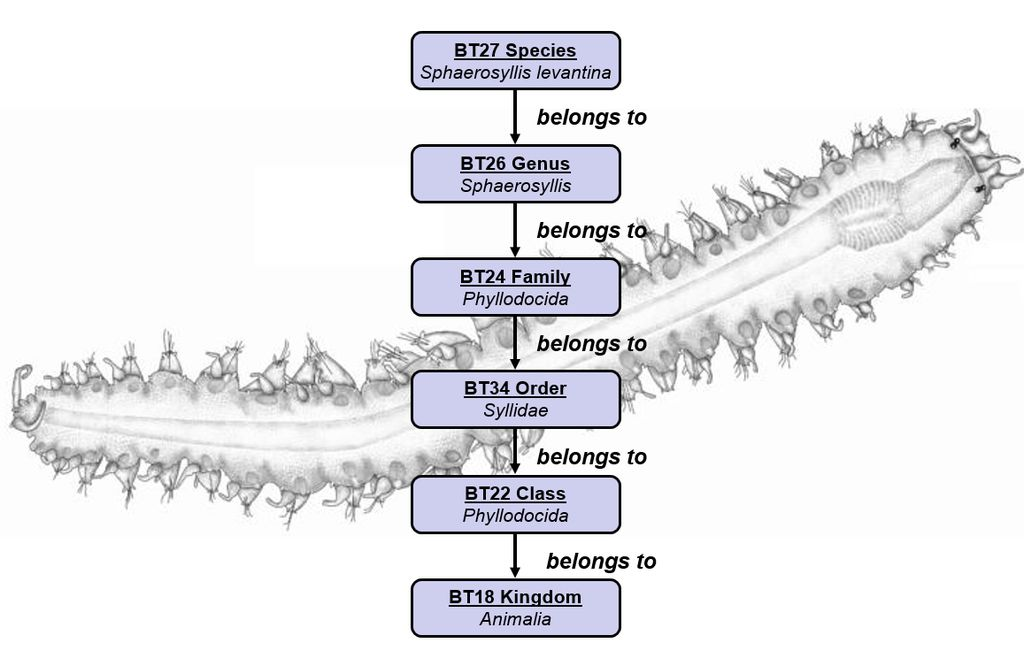
Taxonomy Conceptual Modeling Example

**Figure 3.**
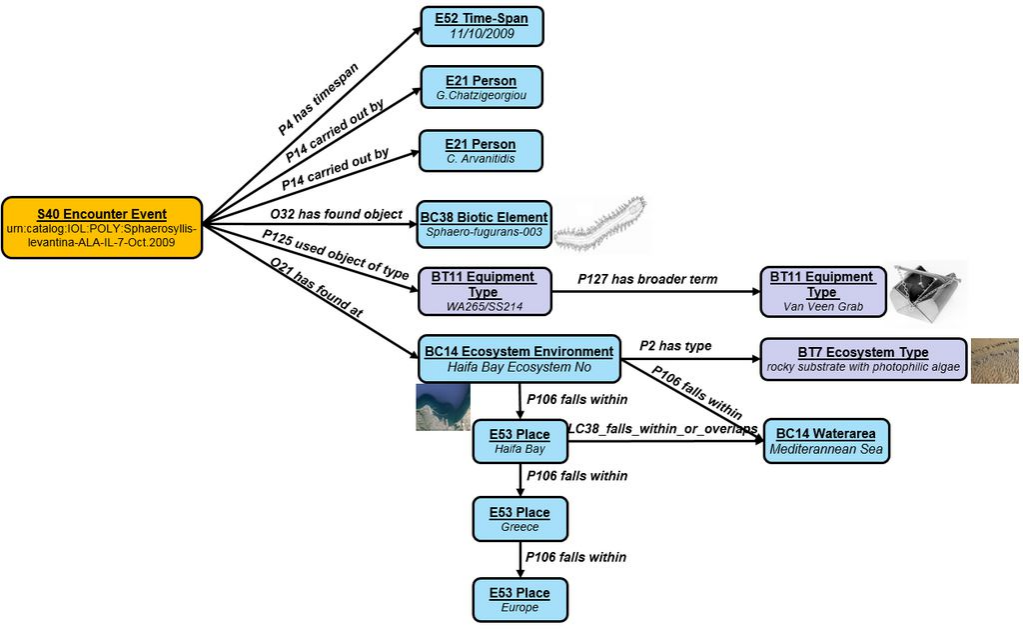
Occurrence Event Conceptual Modeling Example

The next step was the definition of the mappings between the source’s schemata and the centralized schema of the infrastructure. Table 1 shows an example of a high level mapping between DarwinCore (DwC) and the central semantic model of the infrastructure. It is evident from the example that our modelling is more expressive and allows preserving more information about the events. A few modeling examples of some meaningful biodiversity concepts (i.e. species taxonomy and occurrence) are shown in Fig. 2 and Fig. 3.

Furthermore, in rich semantic networks, where the information is constructed using a schema of high complexity, useful deductions are created. In such semantic networks that provide a great level of detail a keyword based querying system or a system based on explicitly defined relationships would be insufficient. Using a specific keyword or just a flat relationship for querying would result in low recall rates, as the system would ignore all the essential information that is hidden behind deductions of relationships linking and reasoning. The proposal was not to simplify the schema for constructing the semantic network, but the schema for querying the complex semantic network. To realize this, we modeled the biodiversity domain in 5 fundamental categories and we defined certain generic fundamental relationships:

***1. Thing*** *(Specimens, Individuals, Publications etc)*

***2. Actor*** *(Scientists, Authors, Institutions ect)*

***3. Place*** *(EcosystemEnvironment, Country, WaterArea etc)*

***4. Event*** *(Identification, Occurrence, Scanning etc)*

***5. Dimension*** *(Length, Weight, Salinity etc)*

**Design Phase**

The first step of the design phase was the design of the architecture, taking into account that the main goals of the data services of the infrastructure are i) the discovery of registered resources within an information community and ii) the return of information that allows a user to locate and access the resource and its curator/creator. This is a tricky task since several issues may occur; for instance resources might not be available online (technical failure/access rights/data policies), or they might be widely distributed/stored or the information of how to access the dataset/collection might be incomplete or missing. In order to achieve this goal, to fulfill the functional requirements and to overcome these issues a new prototypical architecture comprising of 3 main components was designed and implemented.

**Figure 4.**
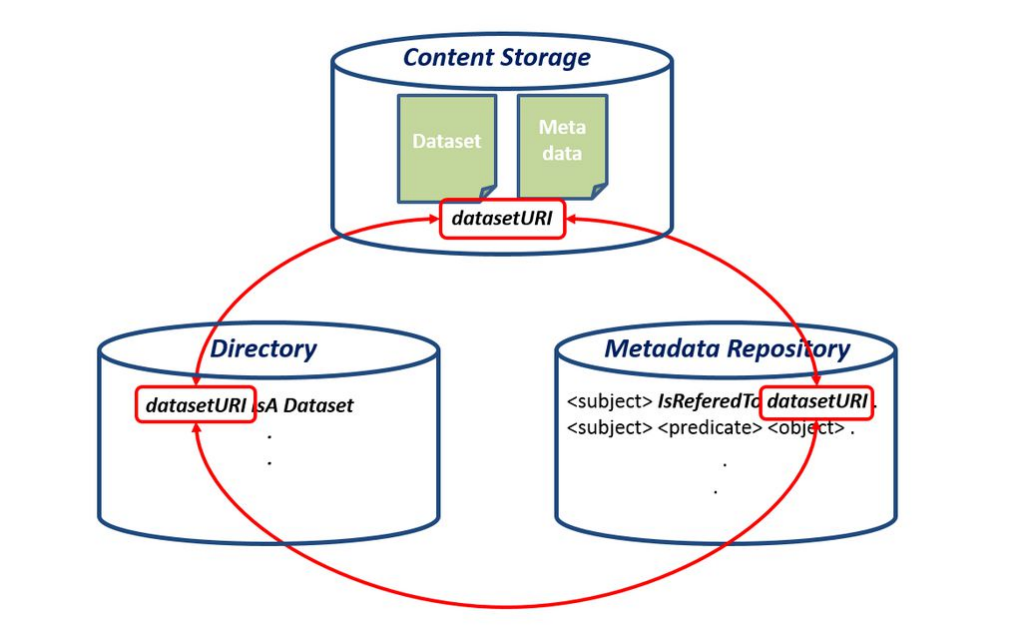
3 Main Components Contents linking through the Dataset's URI

The 3 main components are the **Directory**, the **Metadata Repository** and the **Content Storage** and they can operate independently of each other, and provide different levels of querying capabilities. They are loosely-coupled complements and are linked exclusively by the URI of the results achieving at the same time conceptual and semantic linking and functionality independence (Fig. 4). Also, by adopting this architecture we achieve: (a) a separation between domain specific and domain-independent information, (b) the ability to answer queries even if one of the components fail and (c) the ability to re-create the components in case of failure.

The role of the Directory is to support the discovery of registered resources within an information community and return information that allows a user to locate and access the resource and its curator/creator. Specifically it provides the means to create, edit, update, search information about providers, collections (datasets) and the means to communicate and access with them. The Directory holds information that are independent of the domain. Some Indicative queries that can be answered by the Directory are the following:

- *Which institution hosts the dataset with title “Thunnus Occurences” and how can I have access to it*?
- *Return all the datasets that have been created from HCMR*.

**Figure 5.**
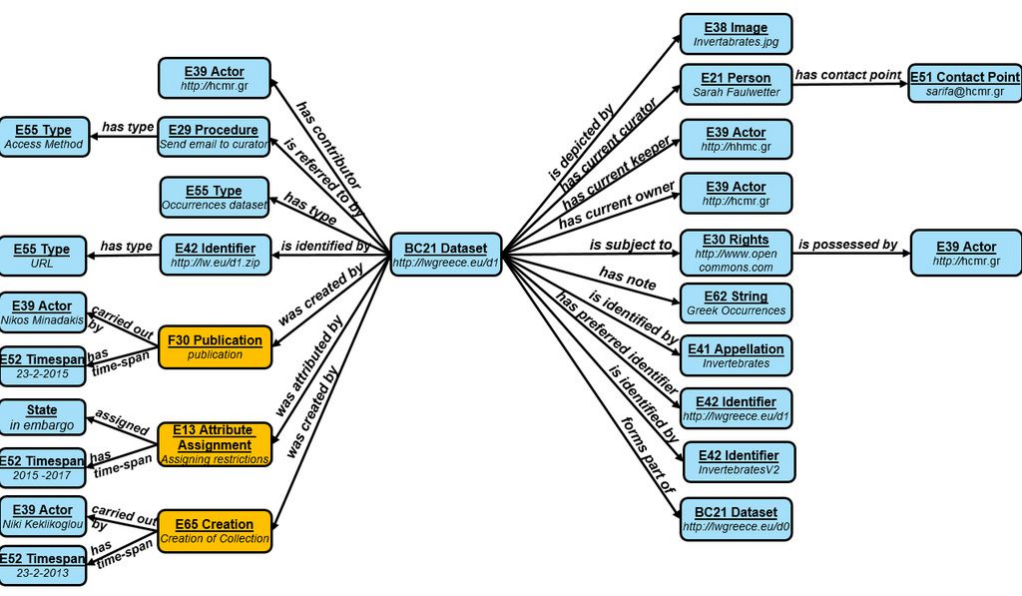
Directory Schema

The data are physically stored in a triple store. An example illustrating part of the schema of the Directory is shown in Fig. 5.

The Metadata Repository is domain dependent and assists dataset discovery by modelling in a rich semantically way part of them. It is also responsible for storing, updating and querying the metadata that the providers would like to be public. It aids information integration and inference. All the metadata that are provided by the data providers are stored into the metadata repository, after they are mapped to the centralized schema of the infrastructure. Furthermore, the semantic models, the inferred triples, the materialization rules products and the instance matching products are stored in the Metadata Repository. The queries that are executed by the users exploit the Metadata Repository and return the datasets that contain the related information. Some indicative queries that can be answered by the Metadata Repository are:

- *Find all datasets about scans depicting marine species.*
- *Find all datasets about species that have been originally described from Greece*

The Content Storage stores any raw data / datasets that the data providers upload to the system (i.e. datasets, images, DNA sequence files, analysis products, etc.). The raw data are linked with the metadata into the Metadata Repository and the Directory entries by using the dataset’s URI.

**Figure 6.**
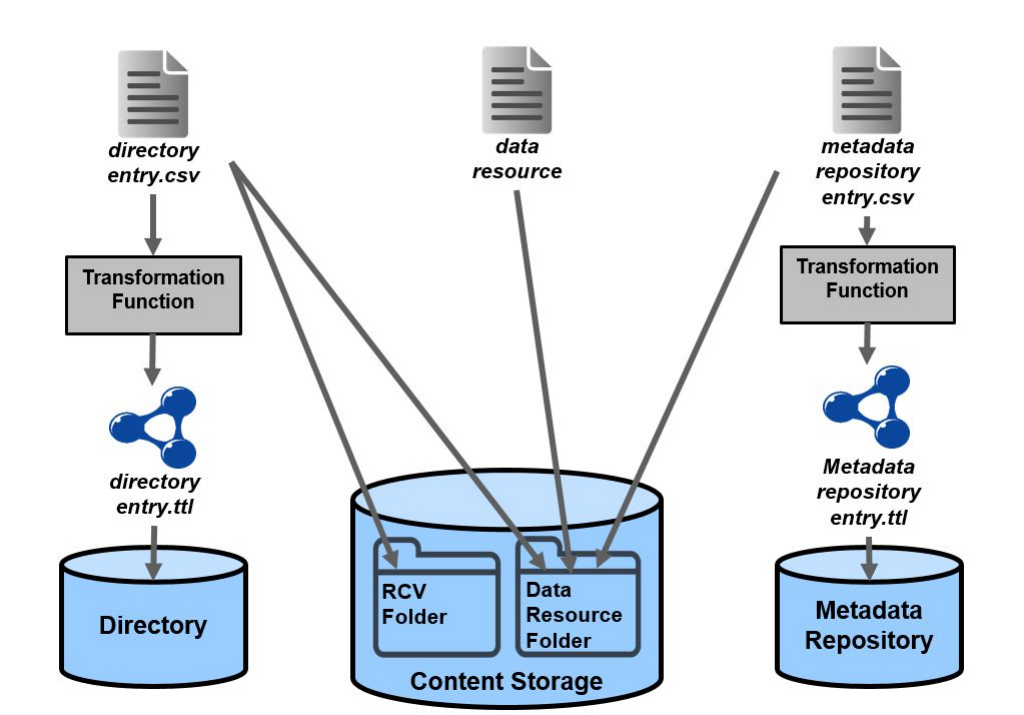
Data Publishing Policy Diagram

An important step that followed the design of the main components was the definition of the policies regarding some important processes such as updating, recovering from failures etc. Most of these policies were designed from scratch for the needs of LifeWatch Greece and have been applied for the first time in the project. Specifically these policies include the publishing policy, as shown in Fig. 6, the updating policy, the versioning policy, the recovery policy and the URI policies. Regarding the URI creation policy of the infrastructure it was decided that the URIs should be human readable, and to provide information on the type of the entity that they identify. The format of the URIs that are automatically created is:

**www.lifewatchgreece.eu/entity/ + entityType/ + entityID_trimed_inLowerCase**

## Web location (URIs)

Homepage: http://metacatalogue.portal.lifewatchgreece.eu/

## Technical specification

Platform: http://metacatalogue.portal.lifewatchgreece.eu/

Programming language: JAVA

Interface language: HTML5, JSP, Javascript

Service endpoint: http://metadator.her.hcmr.gr/DataServices-middleware/

## Repository

Type: Git

Browse URI: https://github.com/isl/LifeWatch_Greece

## Usage rights

### Use license

Creative Commons CCZero

### IP rights notes

To access the data-services web application the users must register to the lifewatch greece portal.

## Implementation

### Implements specification

**Implementation phase**

**Figure 7.**
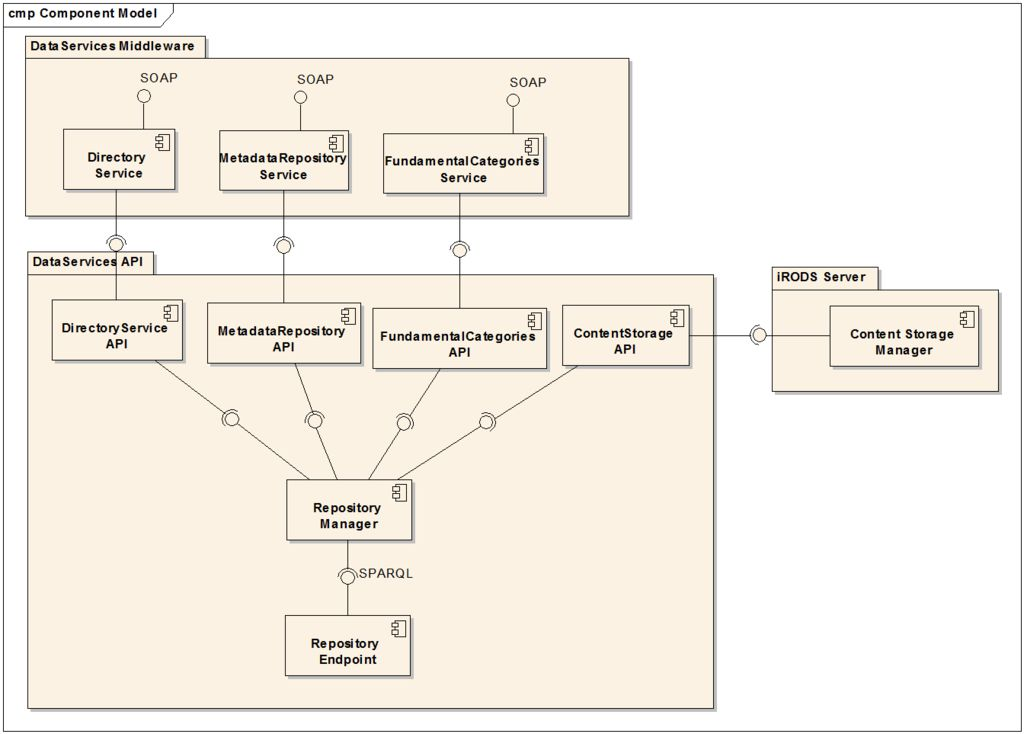
Services Components Diagram

**Figure 8.**
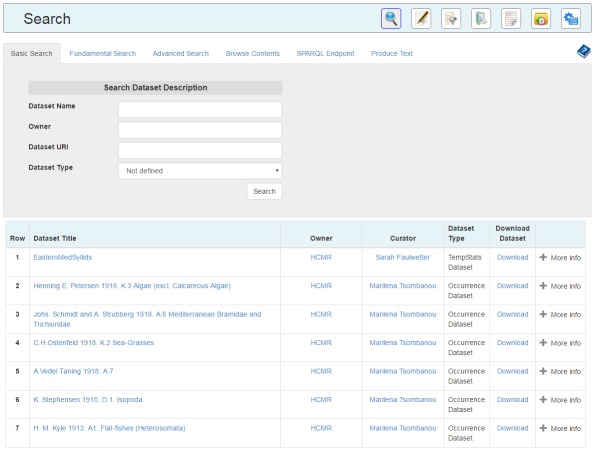
Web Application's GUI snapshots

The first step of the implementation phase was the implementation of the Data Services API and the SOAP Web Services (Fig. 7). These products are the basis of the front-end implementation of the data services infrastructure and are being used for the development of the data services web application (Fig. 8) of the LifeWatch Greece portal and by a number of other e-services and v-labs of the infrastructure. They are publicly available to users and developers and can be reused for the creation of new applications and infrastructures, and also be exploited by the other partners of the LifeWatch Europe Project [Basset and Los 2012].

The main functionalities that are provided by the Data Services API offer the following functions:

***Triple Store Operations***: For the underlying storage of the Directory and the Metadata Repository, we have used a triple store (in our case a Virtuoso triple store). To this end, we have implemented a set of operations to:

a) connect to a semantic triple store

b) import data to the triple store

c) update the triple store contents

d) delete data from the triple store

e) query the triple store

***Biodiversity Data Transformation***: The Data Services API contains a number of functions that transform biodiversity data from CSV format to RDF. The code takes as input specific CSV templates that are filled with biodiversity data, and transforms it to RDF format based on event centric semantic model of the infrastructure. The produced triples can be imported to a triple store.

***Biodiversity Data Querying***: The Data Services API, beside the generic querying functionalities, includes a number of biodiversity domain specific querying functions that return biodiversity data which is stored in triple stores and belongs to one of the main metadata categories. Furthermore, offset and limit parameters can be used to limit the number of returned results enabling, for example, pagination implementations.

***URI Creation***: The API offers three different functions for URI creation. These functions can create URIs that are a concatenation of the prefix, the type of the entity and the id of the entity, or a concatenation of the prefix and the id of the entity, or a hash code.

***Browsing Semantic Graphs***: Two functions for browsing the semantic graphs have been created. The one returns all the outgoing nodes, and their types, of an entity and the other all the incoming nodes, and their types, to an entity. Similar to the querying functions the offset and limit option is provided to limit the number of the returned results.

***Materialization Rules Execution***: In many cases the contents of a semantic graph need to be materialized based on a number of rules. For this reason the Data Services API offers a materialization function that takes as input the SPARQL rules from an external folder and executes them importing the materialized triples to the triple store.

***Textual Description Production***: The Data Services API contains a function that takes as input the Scientific Name of a species and by using standard templates, produces a textual (wikipage like) description, integrating information coming from different datasets and providers.

The web services that were developed to expose the infrastructure functionalities have been packaged into a middleware (the DataServices-middleware) that contains three main services and a number of sub-services. The services use SOAP as a protocol for exchanging messages with clients. The services are:

1. Directory Web Service

- searching [***DirectoryService_Search***]
- adding [***DirectoryService_Insert***]
- updating [***DirectoryService_Update***]
- deleting [***DirectoryService_Delete***]

2. Metadata Repository Web Service

- searching [***MetadataRepository_Search***] adding [***MetadataRepository _Insert***]
- updating [***MetadataRepository _Update***]
- deleting [***MetadataRepository _Delete***]
- browsing [***MetadataRepository _Select***]
- producing text [***MetadataRepository _Text***]

​3. Fundamental Categories Web Service

Besides the main SOAP services a REST service was developed to allow the annotations of a species URIs using the morphological traits contained in the Polytraits database (Faulwetter et al. 2014).

**Web Application**

To expose to the users of LifeWatch Greece the functionalities of the Data Services, a Web Application (Fig. 8) has been developed using the Data Services API and has been integrated to the portal of the infrastructure.

The providers can publish their data and their metadata through the Data Services Web Application. Moreover, the web application provides a number of search capabilities to the users. These are the *Basic Search*, which is actually a search on the *Directory*, the *Advanced Search* which gives the capability to the users to search on the Metadata Repository by selecting a metadata category, the *Fundamental Search*, which exposes the Fundamental Relationships/Categories querying mechanisms, the *Browsing* which takes as input a URI and enables a semantic graph navigation and the SPARQL querying execution on the SPARQL endpoint.

Almost every result that is returned by the search functions is a link. These links are created by using the unique identifiers that we produced and by using a number of browsing functions that we implemented. By clicking the link someone can find more information about the entity and navigate the semantic graph.

**Figure 9.**
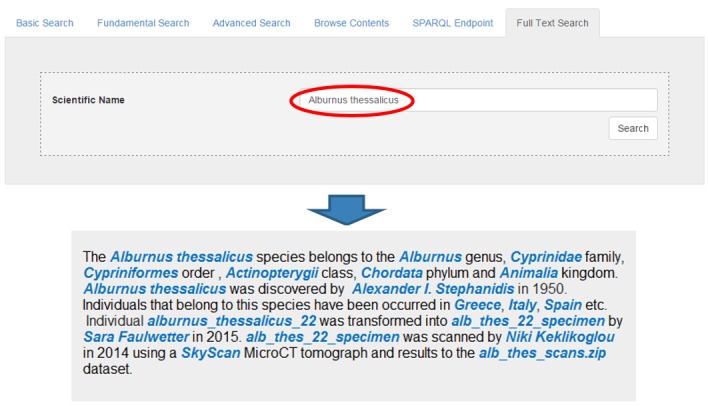
Natural Language production by using RDF templates

By using standard templates it was made possible to return information about a specific concept in natural language (Fig. 9). The user can insert a scientific name and take back as a story or as a Wikipedia entry the aggregated information. All this text is produced automatically. For example if a user enters the name "*Alburnus thessalicus*" the result that is shown in Fig. 9, will be returned. Every link is an entity that is extracted by the underlying triple stores and is clickable allowing browsing on the semantic graph.

**Figure 10.**
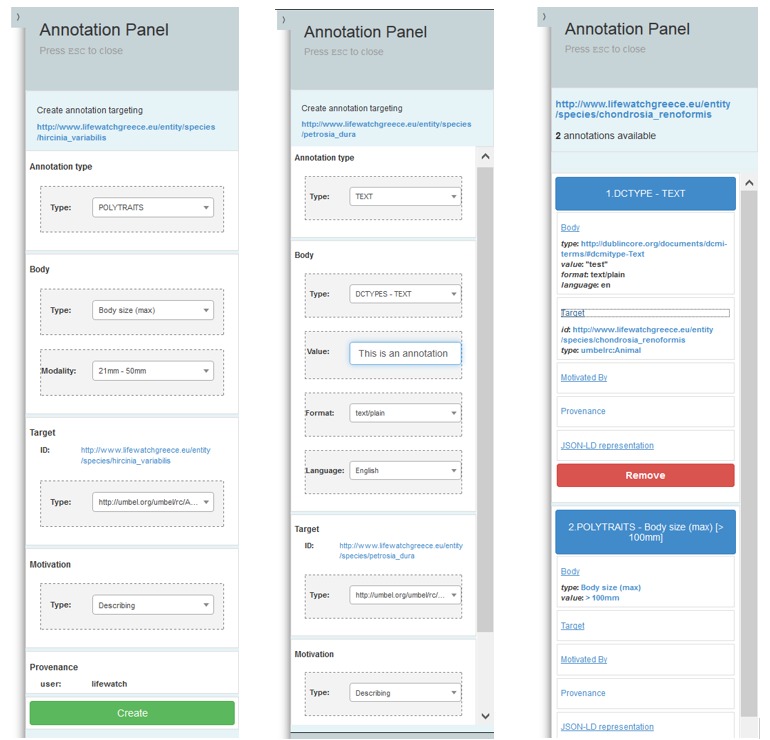
Annotation Service GUI

Furthermore, users are able to create, retrieve and delete Polytrait and free-text annotations over the available species URIs through special widgets that can be activated through a button in the results page of the Advanced Search > Occurrence Search Type facility. When the annotation mode has been activated all the available species URIs can be annotated and their respective annotations can be retrieved/deleted (Fig. 10).

To assist the administrator with tasks such as the managing of the infrastructure’s main components, configuring the system, recovering from failures, materializing the triple stores, and furthermore to make the infrastructure as generic as possibly, decoupled from the underlying components, a number of pages visible exclusively to the administrators have been included in the web application.

The Data Services web application that has been integrated in the LifeWatch Greece portal have been populated with datasets and metadata coming from the greek biodiversity community. This was the main scalability and functionality test of the Data Services. Specifically the infrastructure, that is continuously being populated, contains information about almost:

- 100 datasets
- 700 Occurrence Events
- 100 Identification Events
- 100 Micro CT Scannings
- 100 Micro CT Reconstructions
- 50 Micro CT Posprocessings
- 100 Specimens
- 30 Environmental Measurement Events
- 200 Common Names
- 100 Scientific Names
- 200 Taxonomic Classifications
- 170 Synonyms

and a small number of genetic sampling and sequencing events, occurrence statistics, statistical evaluations, morphometric measurements, morphological characteristic assignments and specimen collections.

**Information Integration Example**

The following example demonstrates the innovative work done in LifeWatch Greece by exploiting the Data Services capabilities to discover data resources. The scenario includes a user that searches for datasets that contain information about occurrences of species in the Mediterranean Sea.

**Figure 11.**
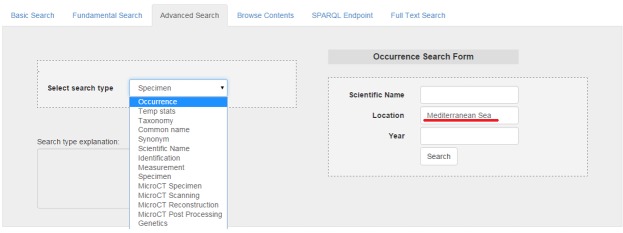
Selection of Occurrences of Species in the Mediterranean Sea

The first action of the user is the selection of occurrence metadata category on the *Advanced Search* form and the filling of Mediterranean Sea in the location field (Fig. 11).

**Figure 12.**
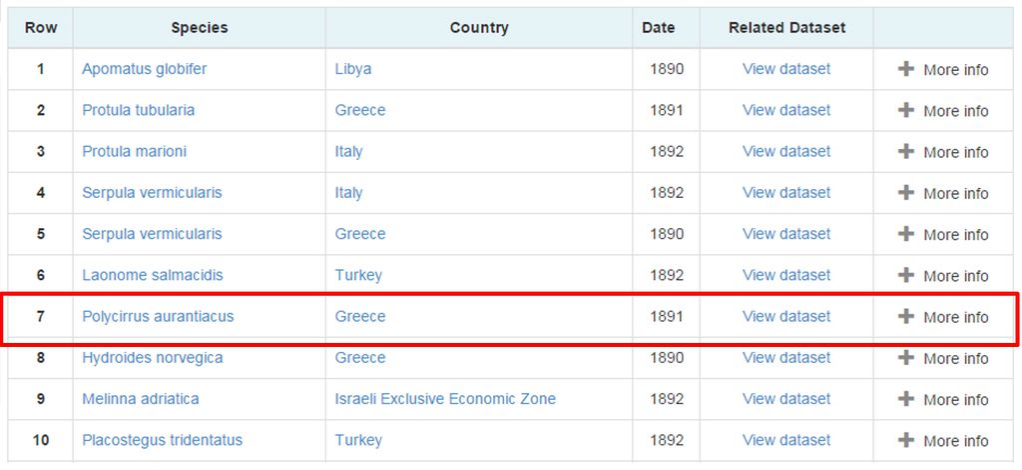
Occurrences in the Mediterranean Sea Search Results

A list of all the occurrence events that took place in Mediterranean Sea are returned (Fig. 12).

**Figure 13.**
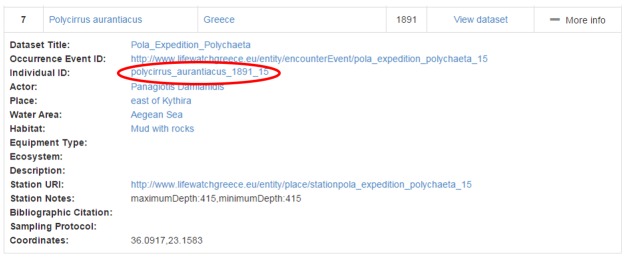
Occurrence’s Complete Information List

We suppose that the user is interested in the "*Polycirrus aurantiacus*" occurrence event so by clicking "more info", more information about the event is returned to the user. By clicking on the view dataset the user will be redirected to the directory entry, find more information about the dataset that this event is described to and download it or access the curators/creators (Fig. 13).

**Figure 14.**
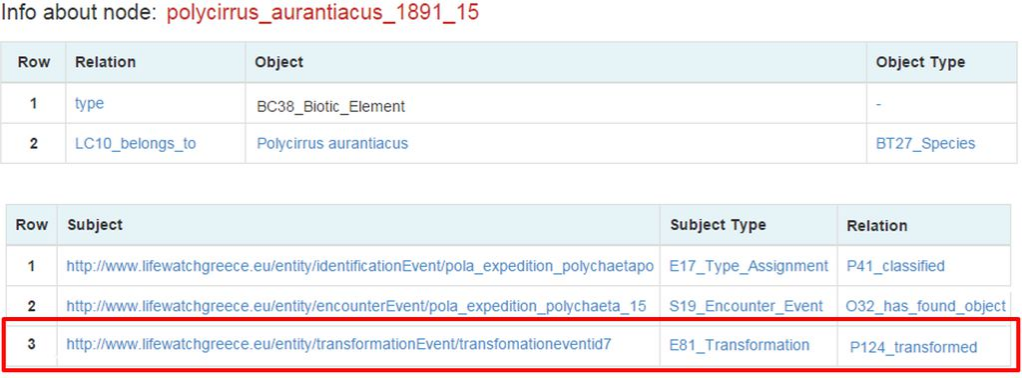
Polycirrus_aurantiacus_1981_15 browsing page

We further suppose that the user is also interested in the individual that was found during the occurrence event. So by clicking on its identifier (Polycirrus_aurantiacus_1981_15) the user is being directed to the entities browsing page (Fig. 14).

**Figure 15.**
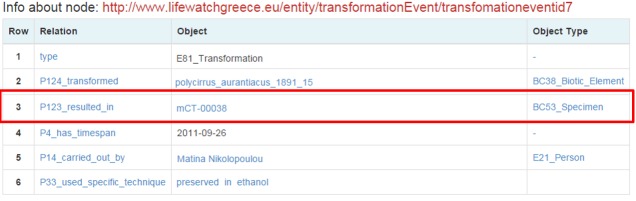
Transformation of Polycirrus_aurantiacus_1981_15 event

More data related to the individual is returned, such as other events that it participated. But the user is interested in a transformation event that the individual had participated. By clicking it the user is directed to a page about this event (Fig. 15).

The user notices that the individual was transformed into a specimen by using a preservation in ethanol method and the new name is mCT-00038.

**Figure 16.**
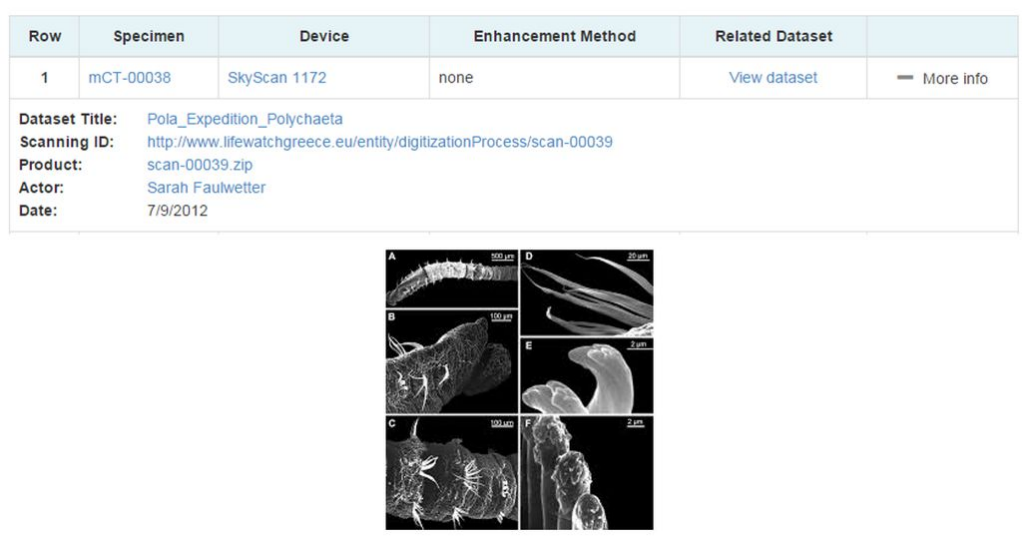
Micro-CT scanning's information and kept metadata.

By clicking on the specimen’s id the user finds out that it was scanned by a Skyscann MicroCT tomograph by Sarah Faulwetter in 2012 and a dataset was produced. The user can retrieve information about this dataset and download it (Fig. 16).

Summarizing, the user initially desired to find datasets that contain information about occurrence events of species in the Mediterranean Sea. He was able to easily discover the requested information but also by browsing the semantic network the user was able to find microCT scannings about the species of interest that come from a different institution, dataset and time, that the user was probably not aware of. This was just a simple example of information integration, navigation to the semantic graph, unique identifier assignment and efficient discovery of dataset that we achieve in LifeWatch Greece data services.

**Technologies**

The Data Services API has been developed in JAVA. The Web Services follow the SOAP and REST protocols while the Web Application is mainly developed using JAVA Servlets, JSP, JQuery and Bootstrap, deployed on a Glassfish Server.

RDF [Erling and Mikhailov 2009] has been chosen for the data representation and the semantic models have been implemented in RDFS and OWL. SPARQL is the main querying language, but also MySQL has been used for the needs of the web application.

OpenLink Virtuoso which is a general purpose RDF triple store with extensive SPARQL and RDF support [8], has been used for the storage needs of the Metadata Repository and the Directory. iRODS [Rajasekar et al. 2010], which is a federated file system, has been chosen for the Content Storage requirements, and iDrop is the administrative user interface that has been used on top of it.

The Fundamental Query User Interface was a results of the tight cooperation of FORTH and Metaphacts and has been implemented mainly in JAVA and ReactJS.

The implementation of the Morphological Annotation REST API uses the anno4j library for accessing the annotations. The implemented annotation services exploit the Blazegraph triple-store.

Finally, Biovel’s Data Refinement Workflow has been used for the Data-Refinement of the datasets.

## Figures and Tables

**Table 1.** An example of a mapping between DwC to LifeWatch Greece semantic model

DwC	**LifeWatch Greece Semantic Model**
occurrenceID	S19 Encounter Event ->P48 has preferred identifier ->E42 Identifier
recordedBy	S19 Encounter Event ->P14 carried out by ->E39 Actor (BT9 Actor Type, BC8 Actor)
